# Grape Seed Proanthocyanidin Ameliorates Cardiac Toxicity Induced by Boldenone Undecylenate through Inhibition of NADPH Oxidase and Reduction in the Expression of NOX2 and NOX4

**DOI:** 10.1155/2018/9434385

**Published:** 2018-07-05

**Authors:** Ehab Tousson, Rehab Mohmed Elgharabawy, Thanaa Ahmed Elmasry

**Affiliations:** ^1^Department of Zoology, Faculty of Science, Tanta University, Tanta, Egypt; ^2^Department of Pharmacology & Toxicology, Faculty of Pharmacy, Tanta University, Tanta, Egypt; ^3^Department of Pharmacology & Toxicology, Faculty of Pharmacy, Qassim University, Buraydah, Saudi Arabia; ^4^Department of Pharmaceutical Science, Faculty of Pharmacy, Princess Nourah Bint Abdulrahman University, Riyadh, Saudi Arabia

## Abstract

The effect of anabolic androgenic steroids on the cardiovascular system is poorly understood. Increased production of free radicals is coupled to the pathophysiology of many alterations within the circulatory system. The only function of the enzyme family NADPH oxidases (NOXs) is the generation of reactive oxygen species (ROS). Therefore, this study investigated the beneficial role of grape seed proanthocyanidin extract (GSPE) in ameliorating cardiac toxicity induced by the anabolic steroid Boldenone in male rats through NOX inhibition and reduction in the expression of NOX2 and NOX4. This study was conducted on forty male rats which are divided into four groups (normal control, positive control or GSPE, Boldenone, and posttreatment Boldenone with GSPE). A significant increase in relative body weight, relative heart weight, and hemodynamic parameters, as well as serum concentrations of lactate dehydrogenase, creatine kinase, creatine kinase-muscle brain, myoglobin, cholesterol, low-density lipoprotein cholesterol, risk factor 1/2, K^+^, and Cl^−^, in treated rats with Boldenone when compared with control. We also noted a significant increase in the levels of cardiac malondialdehyde, H_2_O_2_ generation in heart tissues, mRNA expression of NOX2 and NOX4, and immunoreactivity to proliferating cell nuclear antigen (PCNA). Posttreated rats with Boldenone and GSPE ameliorated cardiac toxicity via inhibition of NOX and a reduction in alteration of the expression of NOX2, NOX4, and PCNA induced by Boldenone. These novel insights into the antioxidative activity of GSPE should serve as a basis for the development of improved chemopreventive or therapeutic strategies for cardiac toxicity.

## 1. Introduction

The performance-enhancing agents are commonly abused by skilled athletes. Among these agents, many have no proven merits and are linked to serious adverse effects [1]. Androgenic anabolic steroids (AASs), such as Boldenone, are abused to enhance muscle mass, strength, and growth as well as to enhance athletic performance. Many countries have forbidden the use of AAS due to their adverse effects [2].

The indiscriminate use of Boldenone for enhanced physical performance and muscular appearance in young people is associated with several harmful side effects. Therefore, Boldenone has been classified as “class 2A” (growth promoter and steroid; probable human carcinogen with a high carcinogenic index) by the International Agency for Research on Cancer [3]. The anabolic steroids adverse effects in men include enlarged breast, inhibition of endogenous testosterone, decreased production of sperms, and atrophy of testes [4, 5]. However, a relatively small number of studies have investigated the effects of anabolic steroids on the circulatory system. Cardiovascular diseases are the leading cause of disability and death worldwide and impose a huge burden on affected individuals and society. In young athletes abusing anabolic steroids, acute myocardial infarction may occur without any past history of heart disease.

Heart pathophysiology is characterized by the alteration in the redox signaling xanthine oxidase, cytochrome P-450, or the mitochondrial electron transport chain as a byproduct, or directly by the NADPH oxidase (NOX) family of enzymes plays a role in the generation of reactive oxygen species (ROS) [6].

NOX1–NOX5 and DUOX1/2 (NOX family) are expressed differentially between tissues. These enzymes participate in many cellular procedures including the proliferation of cells, the release of calcium, and biosynthesis of hormone; however, their overexpression is linked to the pathophysiology of several diseases [7]. Further, the role of NOXs as generators of ROS is noteworthy as these are the only enzymes in which ROS generation is the primary and only known function. An increasing amount of data has demonstrated clearly that the expression and activity of NOXs correlate with the development and progression of cardiovascular diseases [8]. Antioxidant systems react with intracellular ROS to produce less reactive compounds. Glutathione peroxidase (GPx) and catalase are indulged in hydrogen peroxide detoxification to produce water or in a glutathione- (GSH-) dependent reaction. Superoxide dismutase (SOD) catalyzes the transformation of superoxide to hydrogen peroxide [9].

Recently, several polyphenolic antioxidants derived from grape seeds have been implicated in protection of cell [10]. Extract of grape seed proanthocyanidin (GSPE) is a rich source of proanthocyanidins. The latter are natural antioxidants composed of various polyphenolic compounds with protective effects against ROS-mediated myocardial ischemia-reperfusion injury and apoptosis [11]. Therefore, the biological activities of proanthocyanidins (antioxidant, anti-inflammatory, and anticarcinogenic) and their protective effects (reduction of mitochondrial damage and apoptosis inhibition) [12] have garnered considerable interest.

The important goals of this work were (i) to demonstrate the involvement of NOX2 and NOX4 in oxidative stress in response to Boldenone administration; (ii) to elucidate the role of NOX2 and NOX4 in mediating pathologic hypertrophy in response to Boldenone administration; and (iii) to establish that GSPE exerts ameliorative effects on the endogenous NOX2 and NOX4 expression in the heart, with roles in the regulation of the redox system.

## 2. Materials and Methods

### 2.1. Experimental Animals

This study followed the ethical criteria approved by the Ethical Committee of the National Research Center of Egypt. The Animal Ethics Committee of the Faculty of Science, Tanta University (Tanta, Egypt), provided an approval to the protocol of this study.

This study was conducted on 40 male albino Sprague*–*Dawley rats (100–110 g; 7-8 weeks) obtained from Tanta Alhelow Center, Tanta, Egypt. Animals were housed in an environmentally controlled room with lighting (12 h light-dark cycle) and temperature (22–25°C) and had free access to food and water. Close monitoring of the animals was done during the treatment period (8 weeks). The water intake, food intake, and body weights were recorded every week throughout the experimental period.

Rats were divided into four equal groups (10 each) after 2 weeks of acclimatization: normal control (administered vehicle (sesame oil) injection); positive control or GSPE (GSPE was administered via a stomach tube at 50 mg/kg body weight, twice a week) [11]; Boldenone (treated with Boldenone undecylenate (5 mg/kg/week, i.m.)) [13]; and Boldenone then posttreatment with GSPE (treated at the doses and routes mentioned above). Boldenone and vehicle were injected in the hind limbs for 8 weeks.

Animals were decapitated at the end of the study period, after 12 h of fasting. Trunk blood was collected immediately and placed in nonheparinized glass tubes. The blood samples were centrifuged at 3000 ×g for 15 min. Serum was collected and stored at −20°C in a clean, stoppered plastic vial until analyses of serum parameters. The heart was removed, cleaned carefully in cold physiologic (0.9%) saline, and weighed. Calculation of the relative heart weight (RHW) was done using the following equation:

(1) $$\begin{array}{c} RHW=heart\;weight\times \frac{100}{body\;weight}. \end{array}$$

The heart was cut immediately from the base to the apex to make transverse slices of the ventricles. The halves of hearts from each group were fixed in 10% neutral buffer formalin for histology and immunohistochemical (IHC) examination, and the remainders were stored at −80°C for analysis of oxidative stress parameters.

### 2.2. Chemicals and Reagents

Boldenone undecylenate (EQUI-GAN®) vials were obtained from Laboratorios Tornel (Méx, Mexico). GSPE (USP-1298208) was purchased from Sigma–Aldrich (Saint Louis, MO, USA).

### 2.3. Hemodynamic Studies

A miniature pressure transducer (Mikro-Tip®; Millar, Houston, TX, USA) was introduced into the right carotid artery until reaching the left ventricle after anesthetizing animals. Sodium pentobarbital in a dose of 50 mg/kg i.p was used. Aortic diastolic pressure (ADP), aortic systolic pressure (ASP), left ventricular end-diastolic pressure (LVEDP), and left ventricular peak systolic pressure (LVPSP) were recorded on a personal computer using the Axotape data-acquisition program. After hemodynamic recordings, rats were killed and the hearts removed for additional studies [14].

### 2.4. Measurement of Cardiac Biomarkers

Serum lactate dehydrogenase (LDH) activity was measured by a kinetic method using kits (Vitro Scient, Cairo, Egypt) according to a method described by Whitaker [15]. The level of creatine kinase (CK) in serum was determined by an akinetic method using kits (Vitro Scient) according to the method described by Zilva and Pannall [16]. Creatine kinase-muscle brain (CK-MB) activity in serum was determined using an assay kit (BioAssay Systems, Hayward, CA, USA) based on the method of Bishop et al. [17]. The myoglobin concentration in serum was assayed using a kit (Reactivos Spinreact, Girona, Spain) according to the method of Müller et al. [18]. The cardiac troponin T level in whole blood was measured using a Cobas® h 232 immunoassay analyzer (Roche Diagnostics, Mannheim, Germany) at a detection range of 0.1–3 *μ*g/L.

### 2.5. Measurement of the Biomarkers of Cardiac Oxidative Stress

Heart tissues were weighed, and Potter–Elvehjem-type homogenizer was used for homogenization by adding potassium phosphate buffer (pH 7.4) and ice-cold 1.15% KCl-0.01 mol/L sodium to the heart tissues. The supernatant was obtained by centrifugation of homogenate at 10,000 ×g for 20 min at 4°C, and the resultant supernatant was used for analysis.

Malondialdehyde (MDA) was detected by analyses of thiobarbituric acid-reactive substances and measured as reported by Buege and Aust [19]. The content of reduced GSH in heart homogenates was measured using the method of Ellman [20]. SOD activity in heart homogenates was assayed according to the method of Misra et al. [21]. Catalase catalyzes the conversion of H_2_O_2_ to water. Catalase activity in tissue supernatants was detected using a spectrophotometer, and the absorbance was recorded at 240 nm by calculating the rate of degradation of H_2_O_2_. Catalase activity was measured as unit/mg protein [22].

### 2.6. Measurement of H_2_O_2_ Generation

H_2_O_2_ generation in the heart was measured by determining the production of a fluorescent-oxidized product as described previously by Fortunato et al. [23]. Fluorescence was determined at 30°C using a microplate fluorescence reader at an emission wavelength of 595 nm and an excitation wavelength of 530 nm. The variation between the activity in the presence and absence of NADPH determines the activity of NOX. The results were expressed as nanomoles of H_2_O_2_ per hour per milligram of protein (nmol·h^−1^·mg^−1^). Bradford method was used for determination of the protein concentration [24].

### 2.7. Measurement of Total Reduced Thiols

Reduced thiols of heart tissues were analyzed using a spectrophotometer (U-3300; Hitachi, Tokyo, Japan) with 5,5-dithionitrobenzoic acid (DTNB) as described previously by Ellman [20]. The results were obtained at 412 nm and expressed as nmol of reduced DTNB/mg protein [20].

### 2.8. Real-Time Polymerase Chain Reaction (PCR)

RNeasy® Fibrous Tissue Mini Kit (Qiagen, Valencia, CA, USA) was used to extract the total RNA from heart tissues. Real-time PCR was used after DNAse treatment and reverse transcription, as described previously [25]. The internal control used was glyceraldehyde 3-phosphate dehydrogenase. The specific oligonucleotides were obtained from Applied Biosystems (Foster City, CA, USA). The pairs of primers used for RT-PCR were as follows: NOX2: forward: AACTGGCTGTACTGCTTG, reverse: CGAGTCACAGCCACATACAG; NOX4: forward: TCCATCAAGCCAAGATTCTGAG, reverse: GGTTTCCAGTCATCCAGTAGAG; GAPDH: forward: TGATTCTACCCACGGCAAGT, reverse: AGCATCACCCCATTTGATGT.

### 2.9. Measurement of Lipid Profiles

The serum concentration of cholesterol was estimated using a reagent kit (Reactivos Spinreact) according to the method described by Deeg and Ziegenohrm [26]. The serum level of triglycerides was determined using a reagent kit (Reactivos Spinreact) according to the method described by Fossati and Prencipe [27]. The serum level of high-density lipoprotein cholesterol (HDL-C) was analyzed according to the method reported by Norbert [28]. Serum very-low-density lipoprotein cholesterol (vLDL-C) and low-density lipoprotein cholesterol (LDL-C) levels were calculated following the equation of Friedewald et al. [29]:

(2) $$\begin{array}{c} LDL‐C=\frac{total\;cholesterol–triglycerides}{5–HDL‐C}. \end{array}$$

### 2.10. Measurement of Electrolyte Levels

Serum levels of K^+^, Na^+^, Ca^2+^, and Cl^−^ were determined using kits (Sensa Core, Mumbai, India).

### 2.11. Histopathology

Hearts from rats of all studied groups were extracted, washed in physiologic saline, and fixed in 10% formalin. Tissues were dehydrated by using different concentrations of alcohol then replacing the alcohol by xylene. Tissues were embedded in molten paraffin wax. Rotary microtome was used to obtain sections (thickness, 7 *μ*M). Sections were mounted on clean slides. Ehrlich's hematoxylin and eosin were used in staining of the sections [30].

### 2.12. Measurement of Proliferating Cell Nuclear Antigen Immunoreactivity (PCNA-ir)

PCNA-ir was studied according to the method of Tousson et al. [31]. The distribution of PCNA-stained nuclei was analyzed in deparaffinized sections (thickness, 5 *μ*m) using an avidin–biotin–peroxidase IHC method (Elite–ABC; Vector Laboratories, Burlingame, CA, USA) with PCNA monoclonal antibody (1 : 100 dilution; DAKO, Tokyo, Japan).

### 2.13. Statistical Analyses

Data are represented as the means ± SD. Statistical analyses were undertaken using one-way ANOVA to assess significant differences between treatment groups. Differences were considered statistically significant at *p* < 0.01. Statistical analyses were performed using SPSS v21 (IBM, Armonk, NY, USA).

## 3. Results

### 3.1. Effect of Boldenone and GSPE on Weight and Diet Intake

Table 1 shows that food intake, water intake, relative body weight (RBW), and relative heart weight (RHW) in rats injected with Boldenone showed a significant elevation compared with those in the control group. Posttreated rats with Boldenone and GSPE affected the changes in food intake, water intake, RBW, and RHW (Table 1).

### 3.2. Effect of Boldenone and GSPE on Hemodynamics

**Figure 1:**
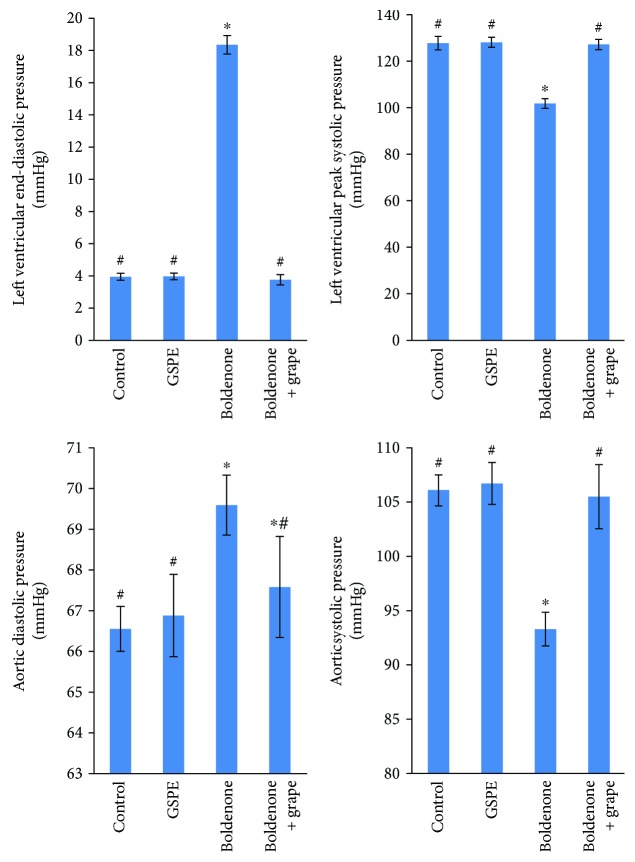
Ventricular and blood pressure in rats treated with GSPE, Boldenone, and Boldenone then GSPE. Value represents mean ± SD of 10 rats. ^∗^Significant difference from the control group at *p* < 0.05. ^#^Significant difference from the Boldenone group at *p* < 0.05.

A significant increase in LVEDP and ADP in rats treated with Boldenone relative to the control group was noted (Figure 1). Further, a significant decrease in LVSP and ASP in rats treated with Boldenone relative to the control group was observed. Posttreated rats with Boldenone and GSPE modulated the observed changes in all hemodynamic data (Figure 1).

### 3.3. Effect of Boldenone and GSPE on Biomarkers of Cardiac Injury

A significant increase in serum levels of LDH, CK, CK-MB, and myoglobin in rats treated with Boldenone compared with those in the control group was noted (Table 2). Insignificant change in the serum level of troponin-T in rats treated with Boldenone alone or in combination with GSPE when compared with the control group was observed (Table 2). A significant decrease in serum levels of LDH, CK, CK-MB, and myoglobin in posttreated rats with Boldenone and GSPE was recorded as compared to Boldenone (Table 2).

### 3.4. Effect of Boldenone and GSPE on Oxidative Stress

**Figure 2:**
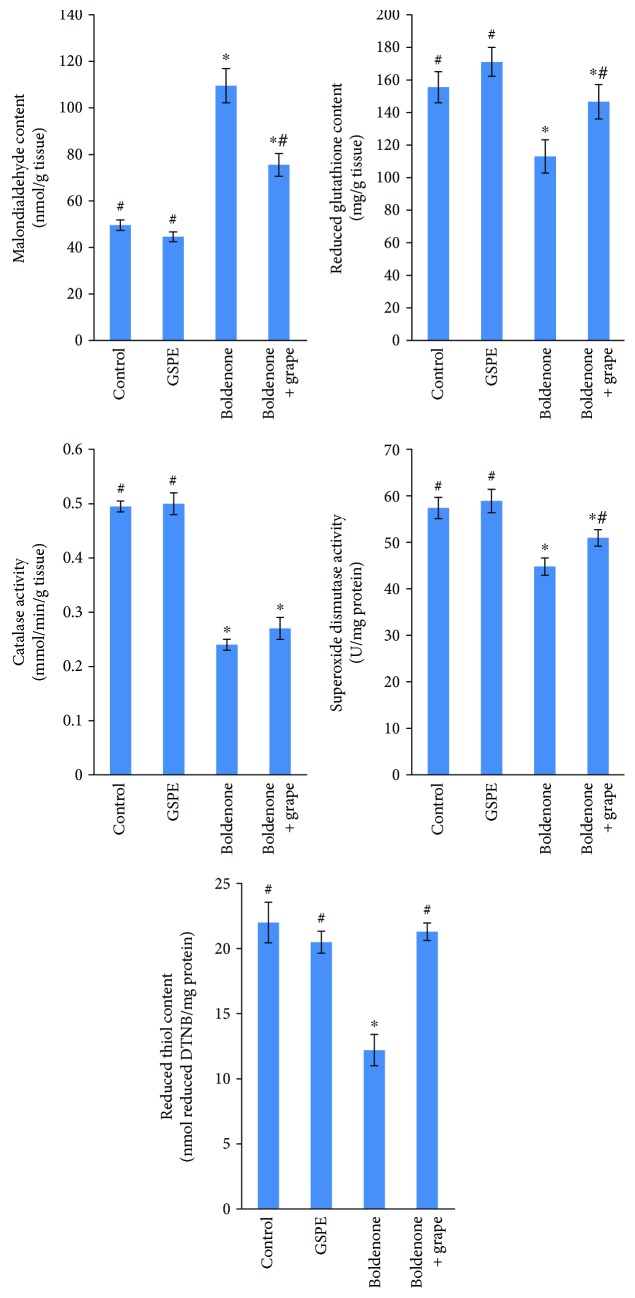
Malondialdehyde content, reduced glutathione content, reduced thiol content, catalase activity, and superoxide dismutase activity in the heart of rats treated with GSPE, Boldenone, and Boldenone then GSPE. Value represents mean ± SD of 10 rats. ^∗^Significant difference from the control group at *p* < 0.05. ^#^Significant difference from the Boldenone group at *p* < 0.05.

A significant increase in MDA levels in the hearts of rats treated with Boldenone compared with those in the control group was observed (Figure 2). A significant decrease in the activity of catalase and SOD as well as levels of reduced thiol content and reduced GSH in the cardiac tissues in rats treated with Boldenone relative to the control group was documented. Posttreated rats with Boldenone and GSPE modulated the changes in all markers of oxidative stress (Figure 2).

### 3.5. Effect of Boldenone and GSPE on NOX Activity and mRNA Levels

**Figure 3:**
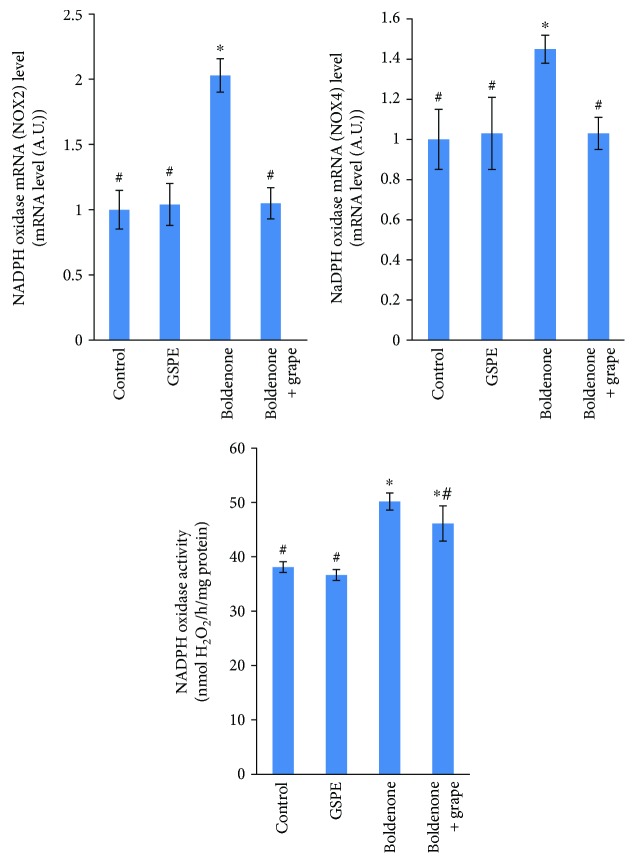
NADPH oxidase activity and NADPH oxidase mRNA levels in the heart of rats treated with GSPE, Boldenone, and Boldenone then GSPE. Value represents mean ± SD of 10 rats. ^∗^Significant difference from the control group at *p* < 0.05. ^#^Significant difference from the Boldenone group at *p* < 0.05.

H_2_O_2_ production was significantly increased in the myocardium of rats treated with Boldenone compared with that in the control groups. A significant decrease in H_2_O_2_ generation in the heart tissues of posttreated rats with Boldenone and GSPE was recorded. To ascertain the source of high generation of H_2_O_2_, the present study studied the mRNA expression of NOX enzymes in the heart. mRNA levels of NOX2 and NOX4 were higher in rat hearts treated with Boldenone compared with those in the control groups. Heart tissues in posttreated rats with Boldenone and GSPE modulated expression of NOX2 mRNA and NOX4 mRNA (Figure 3).

### 3.6. Effect of Boldenone and GSPE on Alterations in Lipid Profiles

A nonsignificant change in serum total lipids, triglycerides, and vLDL-C levels was observed in the Boldenone group (Table 3). A significant increase in the serum levels of cholesterol, LDL-C, risk factor I, and risk factor II in rats treated with Boldenone relative to the control group was observed (Table 3). A significant decrease in HDL-C levels in rats treated with Boldenone relative to the control group was recorded. Posttreatment of rats with GSPE modulated these changes in lipid profiles (Table 3).

### 3.7. Effect of Boldenone and GSPE on Alterations in Electrolyte Levels

Serum levels of K^+^ and Cl^−^ in rats treated with Boldenone were significantly increased compared with those in the control group (Table 4). A significant decrease in serum levels of Na^+^ in rats treated with Boldenone compared with those in the control group was noted (Table 4). A nonsignificant change in serum levels of Ca^2+^in various groups was documented. Posttreatment of rats with GSPE revealed a significant reduction in serum levels of K^+^ and Cl^−^ and a significant elevation in serum levels of Na^+^ (Table 4).

### 3.8. Effect of Boldenone and GSPE on Cardiac Tissues

**Figure 4:**
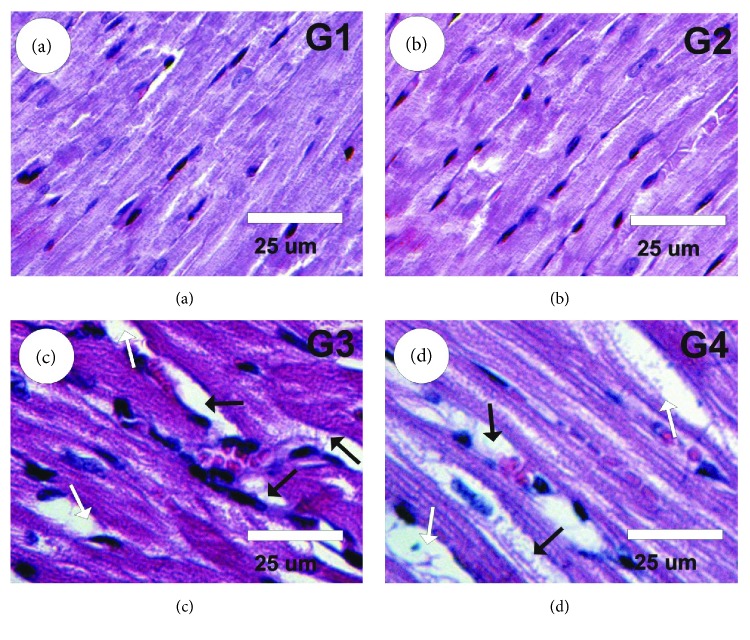
Photomicrographs of rat heart sections stained by HE. (a, b) Heart sections in control (G1) and GSPE (G2) groups revealed normal myofibrillar structure with striations. (c) Heart sections in rats treated with Boldenone (G3) showed severe myocardial lesions due to marked myocardial hypertrophy (white arrows), necrosis, marked interstitial fibrosis (black arrows), misshapen nuclei, moderate focal hemorrhage, and moderate infiltration of leukocytes. (d) Heart sections in posttreated rats with Boldenone and GSPE (G4) exhibited moderate myocardial hypertrophy (white arrows) and interstitial fibrosis (black arrows).

Examination of heart sections under light microscopy in the control and GSPE groups revealed normal myofibrillar structure with striations (Figures 4(a) and 4(b)). Heart sections in rats treated with Boldenone showed severe myocardial lesions due to marked myocardial hypertrophy, necrosis, marked interstitial fibrosis, misshapen nuclei, moderate focal hemorrhage, and moderate infiltration of leukocytes (Figure 4(c)). Heart sections in posttreated rats with Boldenone and GSPE exhibited mild myocardial improvement as moderate myocardial hypertrophy, interstitial fibrosis, and leukocyte infiltration (Figure 4(d)).

### 3.9. Changes in PCNA expression

**Figure 5:**
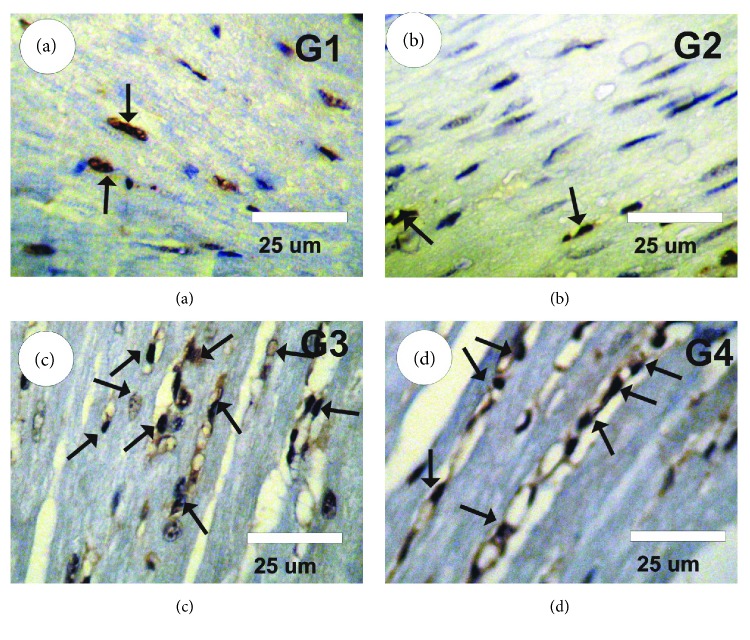
Photomicrographs of heart sections stained with PCNA-ir in the different groups. (a, b) Faint positive reaction (arrows) for PCNA-ir in control (G1) and GSPE (G2) groups. (c) A strong positive reaction (arrows) for PCNA-ir in heart sections of rats treated with Boldenone (G3). (d) PCNA reactivity was decreased significantly (moderate positive reaction for PCNA-ir in treated rats with Boldenone and GSPE) (G4).

The measurement of PCNA-ir in the heart tissues of various groups is shown in Figures 5(a)–5(d). A faint positive reaction for PCNA-ir was observed in the control and GSPE groups (Figures 5(a) and 5(b)). A strong positive reaction for PCNA-ir was detected in myocardium sections of rats treated with Boldenone (Figure 5(c)). PCNA reactivity was decreased significantly (moderate positive reaction for PCNA-ir) following treatment of rats with Boldenone and GSPE (Figure 5(d)).

## 4. Discussion

The anabolic steroid Boldenone is used to enhance the growth of food-producing animals. Boldenone functions by stimulating receptor molecules in muscle cells resulting in the activation of specific genes, leading to protein production [32].

The present study revealed that intramuscular injection of Boldenone in male rats induces a significant elevation in food intake, fluid intake, RBW, and RHW. Tousson et al. [13] demonstrated that the RBW of male rabbits increased significantly following Boldenone injection. Shabir et al. [33] reported a significant increase in food intake, water intake, and weight gain in male rats after Boldenone injection.

Among the various documented toxic and hormonal effects of AASs, the cardiovascular effects of these drugs require closer examination. AASs have two distinct effects: anabolic (promotion of cell growth) and androgenic (enhancement of masculine characteristics). The anabolic effects of AASs lead to increased cellular protein synthesis, resulting in a buildup of muscles. AASs exert their effects on cardiomyocytes through androgen receptors, leading to hypertrophy and dilation, as well as altered relaxation and contraction of the left ventricle. Echocardiographic studies revealed that supraphysiologic doses of AASs induce morphologic and functional alterations in the heart, including a tendency toward myocardial hypertrophy, increase in heart chamber diameter, and alterations in ventricular relaxation and diastolic function [34].

This study revealed that intramuscular injection of Boldenone to male rats elicited a significant increase in the serum levels of LDH, CK, CK-MB, and myoglobin, suggesting muscle damage. Serum levels of CK-MB are used in the discovery of myocardial disorders [35]. Handelsman [36] reported that administration of testosterone and nandrolone to adolescent rabbits induced increases in serum levels of CK-MB. Further, Kerr and Congeni [37] reported that nandrolone injections in rabbits induced increases in serum levels of CK-MB. Lok et al. [38] noted an increase in CK-MB levels in male rats after testosterone injection; however, Tasgin et al. [39] reported no increase in CK-MB levels in female rats. Razmaraii et al. [40] reported that GSPE elicits myocardial protection and vasodilatation *in vivo* and *in vitro*; this finding is in accordance with our results, which showed that rats posttreated with GSPE showed a significant decrease in the serum levels of LDH, CK, CK-MB, and myoglobin.

ROS are generated by tissues all over the body. Continuously, the antioxidant systems scavenge ROS in the cells and convert them to less harmful compounds [10]. ROS attack the biomolecules (DNA, proteins, and lipids) if the capacity of antioxidant functions was reduced. This reaction changes the structure of biomolecules and subsequently impairs the function of cells and genesis of many diseases [41].

We investigated whether Boldenone, an anabolic steroid commonly used by body builders and athletes, interferes with the balance of the redox system in the myocytes. Intramuscular injection of Boldenone in male rats induced changes in the levels of oxidative stress biomarkers and antioxidant defense systems in cardiac muscles. Our results showed a significant elevation in MDA level, as well as a significant decrease in the activity of catalase, SOD, reduced thiol content, and GSH in cardiac muscles after Boldenone injection. Interestingly, ROS and MDA were linked with histopathological changes in all types of muscle injury, mainly in toxic muscle damage caused by drugs. The results of the present study are in agreement with those of El-Moghazy et al. [42], who demonstrated that Boldenone induces oxidative stress in liver and kidney tissues, and those of Ali et al. [43] who found that Boldenone induces oxidative stress in the smooth, cardiac, and skeletal muscles of rabbits.

NOX has received considerable attention as a major cause of oxidative stress leading to vascular disease. Moreover, various NOX subunits play a part in the occurrence of heart failure, stroke, cancer, lung fibrosis, and diabetes mellitus. NOX-derived ROS leads to disease in different ways: for example, spatially confined levels of ROS may interfere with a particular signaling pathway, and high levels (local or systemic) that are directly cytotoxic may cause apoptosis or disrupt redox-sensitive signaling cascades. These systems are in constant interaction with NOX subunits. Due to the complex mechanisms involved in NOX activation, these enzymes may be targeted at several different levels of their activity: (i) decreased NOX expression has inhibitory effects on these enzymes and (ii) NOX activation may be decreased by blocking the translocation of its cytosolic subunits (if present) to the cell membrane [44].

The natural antioxidants including polyphenols have a powerful effect on the inhibition of NOX. A diet rich in polyphenols (green tea, vegetables, fruits, and whole-grain foods) has a beneficial effect in the prevention of circulatory diseases. Polyphenols scavenge superoxide radical and reduce NOX activity in blood vessels and platelets [45]. Moreover, novel polyphenolic compounds that lack typical superoxide-scavenging properties and inhibit NOX directly are currently being investigated. Mansouri et al. [46] reported that GSPE has a beneficial effect on ameliorating lipid metabolism and repairing antioxidant defense systems in the hearts of diabetic rats. Accordingly, GSPE may scavenge free radicals and elicit beneficial effects against Boldenone-induced cardiac damage.

The observation that GSPE has beneficial effects through NOX-inhibitory actions [4] reinforces the therapeutic potential of NOX inhibition for circulatory diseases. Babelova et al. [47] demonstrated that the basal activity of NOX4 may be protective in some clinical situations including cardiac pressure overload and acute ischemia [47].

The present study showed that H_2_O_2_ production was significantly increased in the myocardium of rats treated with Boldenone compared with the control groups. Conversely, a significant reduction in an H_2_O_2_ generation in the myocardium of rats posttreated with GSPE was noted. To evaluate the source of high generation of H_2_O_2_, this study measured the mRNA expression of NOX enzymes in the heart. mRNA levels of NOX2 and NOX4 were higher in rat hearts treated with Boldenone than in those of the control groups. Posttreatment of rat hearts with GSPE modulated the mRNA expression of NOX2 and NOX4.

Gheshlaghi et al. [48] reported that AAS abuse affects myocardial survival and heart function in humans, animal models, and cell cultures [48]. Beutel et al. [49] were the first to study the effects of anabolic steroids on cardiac output. The present study revealed that intramuscular injection of Boldenone in male rats elicited a significant increase in serum levels of cholesterol and LDL-C, which is known to lead to an increased risk of atherosclerotic heart disease and myocardial hypertrophy [50]. In contrast, a significant decrease in the serum level of HDL-C was detected after intramuscular injection with Boldenone compared with that in controls. Serum levels of triglycerides and total lipids did not change between the different groups.

One of the major findings of this study was the relationship between steroid consumption and alteration in lipid profiles. El-Ghareib and Ashry [51] showed that serum levels of cholesterol increased significantly in calves treated with Boldenone compared with those in controls, and our results agreed with their data. However, El-Ghareib and Ashry [51] also found that the levels of total lipid levels increased significantly in calves treated with Boldenone compared with those in controls, but an equivalent observation in rats was not observed in our study. Urhausen et al. [52] noted that HDL-C levels were distinctly lower in athletes who used AASs. Hartgens et al. [50] recorded a decrease in the serum levels of HDL-C, and significant time-based effects were noted for HDL-C. Achar et al. [53] showed that AAS abusers showed a significant increase in the levels of LDL-C and a significant decrease in the levels of HDL-C than nonabusers. Lough et al. [54] reported no effects on plasma concentrations of triglycerides in lambs injected with AASs; these data were in accordance with our results.

This study revealed that administration of Boldenone to male rats induced a significant increase in serum levels of K^+^ and Cl^−^, a significant depletion of Na^+^, but no change in Ca^2+^. In contrast, GSPE treatment improved this alteration in electrolyte levels. A. Demiryurek and S. Demiryurek [55] reported that toxic cardiac concentration of steroids induces inhibition of Na^+^/K^+^-ATPase and its signaling pathways, subsequently sustained to elevate the intracellular level of Ca^2+^ and Na^+^, resulting in cardiac arrhythmias. However, our results are not in accordance with those of Barakat et al. [4], who reported that the intramuscular injection of Boldenone elevated the levels of Na^+^ and Ca^2+^ and decreased those of K^+^ and that propolis modulated these electrolyte changes. The results of the present study are in agreement with those of Yang et al. [11], who revealed that Boldenone induced alterations in electrolyte levels.

Cardiovascular cells express various NOX subtypes. NOX1 is distributed mainly in vascular smooth muscles while NOX2 is distributed in the myocardium. NOX4 is found in vascular smooth muscles, endothelium, and myocardium. In general, NOXs are stimulated by signaling pathways downstream of G protein-coupled receptors like tumor necrosis factor-alpha, angiotensin II, and endothelin 1. Interestingly, NOX activity remains high in NOX2^−/−^ mice after aortic banding, which has been referred to elevated NOX4 expression, indicating that NOX4 may also have a role in pressure-overload left ventricular hypertrophy [56].

Several studies have suggested that NOX activation induces alteration of contractility of hypertrophy myocardium and in heart failure. In animals with LVH, altered coronary endothelial function and impairment of ventricular function were induced by increased myocardial NOX activity [57]. Cheng et al. [58] demonstrated that activation of NOX contributes to increased diastolic stiffness in hypertensive heart failure rats. Antioxidant treatment improves contractile dysfunction of the heart in wild-type animals, suggesting a direct effect of elevated levels of ROS on contractile function [59]. ROS alter the function of the sarcoplasmic reticulum (SR), ryanodine receptor, sarcolemmal ion channels, the SR calcium pump, and contractile proteins. It has also been demonstrated that ROS may alter calmodulin-dependent protein kinase II, possibly via NOX [59].

Overdoses of anabolic steroids cause cardiovascular disorders such as LVH, hypertension, dysrhythmia, coagulation of the blood, altered coronary blood flow, myocardial inflammation, acute coronary inefficiency, cardiac infarction, arteriosclerosis, and cardiac arrest [60]. In our study, histology and IHC results confirmed these biochemical effects. Boldenone administration induced various histologic cardiac lesions in young male rats: marked myocardial hypertrophy, necrosis, marked interstitial fibrosis, misshapen nuclei, moderate focal hemorrhage, moderate infiltration of leukocytes, and increase in PCNA expression were observed.

Boldenone reduces the endogenous biosynthesis (antiandrogenic) and induces steroid biotransformation (estrogenic). These side effects of Boldenone may be attributable to genomic or nongenomic activities (myotrophic). The results of the present study are in agreement with those of Tousson [61], who revealed that Boldenone caused ventricular hypertrophy and fibrosis in the cardiac muscles of male rabbits, and of McCarthy et al. [62], who demonstrated that muscle hypertrophy in skeletal and cardiac muscles in humans was induced by the anabolic steroid. GSPE induced improvement in the ischemic myocardium during reperfusion in rat hearts [63].

## 5. Conclusions

Here, we demonstrated that Boldenone administration disrupts cellular redox balance through NOX activation. Treatment of rats with GSPE ameliorates expression of endogenous NOX2 and NOX4 in the heart and regulated the redox system. These new insights into the antioxidative activity of GSPE should serve as a basis for the development of improved chemopreventive or therapeutic strategies for cardiac toxicity induced by Boldenone.

## Figures and Tables

**Table 1:** Changes in water intake, food intake, relative body weights (RBW), and relative heart weights (RHW) in different groups.

Items	Control	GSPE	Boldenone	Boldenone *+* GSPE
Water intake (mL/rat/day)	31.6 ± 2.44^#^	30.9 ± 1.98^#^	38.9 ± 2.81^∗^	33.0 ± 1.60^∗^^#^
Food intake (g/rat/day)	13.1 ± 1.05^#^	13.2 ± 0.76^#^	18.3 ± 0.95^∗^	13.5 ± 1.01^#^
RBW (g/100 g)	26.8 ± 1.35^#^	24.5 ± 1.22^#^	42.5 ± 3.09^∗^	31.5 ± 2.15^∗^^#^
RHW (g/100 g)	0.51 ± 0.0^#^4	0.50 ± 0.03^#^	0.64 ± 0.02^∗^^#^	0.57 ± 0.04^∗^^#^

Value represents mean ± SD of 10 rats. ^∗^Significant difference from the control group at *p* < 0.05. ^#^Significant difference from the Boldenone group at *p* < 0.05.

**Table 2:** Changes in serum lactate dehydrogenase (LDH), creatine kinase (CK), creatine kinase MB (CK-MB), myoglobin, and troponin-T levels in different groups.

	Control	GSPE	Boldenone	Boldenone *+* GSPE
LDH (U/L)	112.7 ± 8.2^#^	106.5 ± 9.1^#^	139.1 ± 7.8^∗^	123.6 ± 10.5^∗^^#^
CK (U/L)	769.5 ± 11.6^#^	748.8 ± 15.5^#^	955.0 ± 16.5^∗^	817.3 ± 20.8^∗^^#^
CK-MB (ng/mL)	0.202 ± 0.05^#^	0.211 ± 0.11^#^	0.370 ± 0.05^∗^	0.315 ± 0.09^∗^^#^
Myoglobin (ng/mL)	13.8 ± 1.39^#^	13.2 ± 0.76^#^	16.6 ± 0.42^∗^	15.1 ± 1.05^∗^^#^
Troponin T (pg/mL)	0.016 ± 0.06	0.016 ± 0.03	0.015 ± 0.03	0.016 ± 0.05

Value represents mean ± SEM of 10 rats. ^∗^Significant difference from the control group at *p* < 0.05. ^#^Significant difference from the Boldenone group at *p* < 0.05.

**Table 3:** Serum lipid profile levels in different studied groups.

	Control	GSPE	Boldenone	Boldenone + GSPE
Total lipid (mg/dL)	265.5 ± 11.3	261 ± 12.5	264.2 ± 9.4	259.9 ± 10.5
Cholesterol (mg/dL)	103.5 ± 7.06^#^	101 ± 5.40^#^	136.2 ± 9.55^∗^	119.9 ± 6.85^∗^^#^
Triglyceride (mg/dL)	98.2 ± 3.55	92.7 ± 3.95	96.8 ± 6.63	93.6 ± 5.98
HDL (mg/dL)	52.8 ± 3.71^#^	53.5 ± 2.35^#^	43.8 ± 1.66^∗^	47.6 ± 1.88^∗^^#^
LDL (mg/dL)	31.1 ± 1.23^#^	29.7 ± 1.21^#^	73.1 ± 4.15^∗^	53.6 ± 3.59^∗^^#^
vLDL (mg/dL)	19.6 ± 1.50	18.5 ± 1.35	19.4 ± 1.17	18.8 ± 0.93
Risk I	1.96 ± 0.22^#^	1.89 ± 0.14^#^	3.11 ± 0.21^∗^	2.52 ± 0.19^∗^^#^
Risk II	0.59 ± 0.04	0.56 ± 0.04	1.67 ± 0.08^∗^	1.13 ± 0.11^∗^^#^

Value represents mean ± SEM of 10 rats. ^∗^Significant difference from the control group at *p* < 0.05. ^#^Significant difference from the Boldenone group at *p* < 0.05.

**Table 4:** Changes in serum electrolyte ion level in different groups under study.

	Control	GSPE	Boldenone	Boldenone + GSPE
Na+ (mEq/L)	135.8 ± 9.5^#^	136.5 ± 11.1^#^	126.1 ± 11.3^∗^	133.5 ± 8.9^#^
K+ (mEq/L)	3.755 ± 0.34^#^	3.648 ± 0.66^#^	4.480 ± 0.69^∗^	4.228 ± 0.95^∗^^#^
Ca^++^ (mmol/L)	1.222 ± 0.106	1.235 ± 0.08	1.219 ± 0.032	1.228 ± 0.097
Cl^−^ (mmol/L)	100.7 ± 8.30^#^	101.5 ± 8.61^#^	118.5 ± 5.35^∗^	116.5 ± 7.55^∗^

Value represents mean ± SD of 10 rats. ^∗^Significant difference from the control group at *p* < 0.05. ^#^Significant difference from the Boldenone group at *p* < 0.05.

## Data Availability

The data used to support the findings of this study are available from the corresponding author upon request.
